# Promoter Methylation of MLH1, PMS2, MSH2 and p16 Is a Phenomenon of Advanced-Stage HCCs

**DOI:** 10.1371/journal.pone.0084453

**Published:** 2014-01-06

**Authors:** Inga Hinrichsen, Matthias Kemp, Jan Peveling-Oberhag, Sandra Passmann, Guido Plotz, Stefan Zeuzem, Angela Brieger

**Affiliations:** Medical Clinic I, Biomedical Research Laboratory, Goethe-University, Frankfurt a.M., Germany; University of Algarve, Portugal

## Abstract

Epigenetic silencing of tumour suppressor genes has been observed in various cancers. Looking at hepatocellular carcinoma (HCC) specific protein silencing was previously demonstrated to be associated with the Hepatitis C virus (HCV). However, the proposed HCV dependent promoter methylation of DNA mismatch repair (MMR) genes and thereby enhanced progression of hepatocarcinogenesis has been the subject of controversial discussion. We investigated promoter methylation pattern of the MMR genes *MLH1, MSH2 and PMS2* as well as the *cyclin-dependent kinase inhibitor 2A gene* (*p16*) in 61 well characterized patients with HCCs associated with HCV, Hepatitis B virus infection or alcoholic liver disease. DNA was isolated from formalin-fixed, paraffin-embedded tumour and non-tumour adjacent tissue and analysed by methylation-specific PCR. Moreover, microsatellite analysis was performed in tissues showing methylation in MMR gene promoters. Our data demonstrated that promoter methylation of *MLH1*, *MSH2*, *PMS2* and *p16* is present among all considered HCCs. Hereby, promoter silencing was detectable more frequently in advanced-stage HCCs than in low-stage ones. However, there was no significant correlation between aberrant DNA methylation of MMR genes or *p16* and HCV infection in related HCC specimens. In summary, we show that promoter methylation of essential MMR genes and *p16* is detectable in HCCs most dominantly in pT3 stage tumour cases. Since loss of MMR proteins was previously described to be not only responsible for tumour development but also for chemotherapy resistance, the knowledge of mechanisms jointly responsible for HCC progression might enable significant improvement of individual HCC therapy in the future.

## Introduction

Hepatocellular carcinoma (HCC) is one of the most common human malignancies with poor prognosis [1]. Alcohol, aflatoxin, metabolic disorders and chronic infection caused by hepatitis C virus (HCV) and/or hepatitis B virus (HBV) have been defined as the most dominant risk factors for HCC development. Chronic HCV and HBV infections attribute to HCC in more than 80% of the cases all over the world [2]. However, the molecular mechanisms of HCC carcinogenesis seem to be different according to their origins and are still not fully understood. It is known that HCC risk factors induce malignant transformation by increasing cellular turnover as a consequence of chronic liver injuring, regeneration and cirrhosis [3]. This leads to multiple genetic alterations including chromosomal instability with point mutations and deletions causing the activation or inactivation of proto-oncogenes or tumour suppressor genes, respectively. Aberrant epigenetic silencing due to CpG island methylation has emerged as one of the pivotal genetic alterations in HCC development and progression [4], [5], [6].

Hereby, Feng et al. recently hypothesized that HCC resulting from different viral aetiologies is associated with different epigenetic changes [7]. HCV e.g. was demonstrated to effect CpG island methylation pattern especially of those genes responsible for DNA mismatch repair (MMR) [8], [9] and/or the cell cycle regulation [10]. However, neither the methylome analysis of Neumann et al. [11] nor the identification of preferentially HCV dependent methylated genes of Deng et al. [12] verified the proposed relationships. Moreover, Li and co-workers could not detect promoter methylation in some of the proposed HCC associated MMR genes [13] and Wang et al. were not able to show any expression alteration in MLH1 or MSH2 or MSI on 36 tested HCCs [14].

To further clarify the controversial data we first determined the promoter methylation of the three most important MMR genes, MLH1, MSH2 and PMS2 in a European cohort of 61 patients with HCC resulting from different viral aetiologies or alcoholic liver disease. Secondly, we analysed the promoter methylation pattern of the cyclin-dependent kinase inhibitor 2A (p16), a cell cycle regulator gene which was frequently described to be influenced by HCV [10], [15], [16].

## Materials and Methods

### Patients and pathological data

Tumour and non-tumour adjacent tissue was analysed for promoter methylation of MLH1, MSH2, PMS2 and p16 from 61 patients with primary invasive HCC who underwent surgery from 2001 to 2012 at the Goethe University Hospital, Frankfurt, Germany. In 34 of these patients HCC was associated with HCV infection, in 10 patients with HBV infection and in 17 of them with alcoholic liver disease (Table 1). The local ethics committee (University Clinic of Frankfurt, Frankfurt, Germany) approved the study (No. AB-01/2013), and all patients provide their written informed consent to participate in this study. The patient database was anonymized to guarantee privacy. The tissues were formalin-fixed and paraffin-embedded in accordance with standard methods. Histological classification was performed by following the recommendations of the World Health Organization [17].

**Table 1:** Clinical feature of the studied patients.

Serial No.	Age	Sex	Cirrhosis	Grade	Stage	Origin of HCC	Meth. MMR	BAT25	BAT26
**1**	44	m	+	II	pT2, pN0, cM0 R0	HCV	−	ND	ND
**2**	48	m	+	II	pT2, R0	HCV	−	ND	ND
**3**	48	m	+	II	pT2, R0	HCV	+	−	−
**4**	51	m	+	NA	NA	HCV	−	ND	ND
**5**	52	m	+	II	pT3, pNX,L1, V1, R1	HCV	+	T: + NT: +	−
**6**	54	m	+	II	pT4, pN0, R0	HCV	−	ND	ND
**7**	54	m	+	III	pT3, R0	HCV	+	−	−
**8**	55	m	+	NA	NA	HCV	−	ND	ND
**9**	56	m	+	II	pT2, pN0, R0	HCV	−	ND	ND
**10**	56	m	+	NA	ypT1, ypN0, L0, V0, R0	HCV	+	−	−
**11**	59	m	+	II	pT4, pN0, R0	HCV	−	ND	ND
**12**	61	m	+	II	pT1, X, R0	HCV	+	−	−
**13**	61	m	−	II	NA	HCV	+	−	−
**14**	61	m	+	II	pT1, X, R0	HCV	+	−	−
**15**	62	m	+	II	NA	HCV	−	ND	ND
**16**	65	m	−	II	pT3, pN0, L0, V0, R0	HCV	+	T: + NT: −	−
**17**	66	m	+	III	pT3, pN0, V1, L0, R1	HCV	−	ND	ND
**18**	66	m	+	III	pT3, pN0, V1, L0, R1	HCV	+	−	−
**19**	66	m	−	II	pT1, R0	HCV	+	−	−
**20**	68	m	+	I	pT1, R0	HCV	+	T: + NT: −	T: + NT: −
**21**	46	f	+	II	pT1, R2	HCV	+*	T: + NT: −	−
**22**	52	f	+	II	NA	HCV	+*	−	−
**23**	55	f	+	I	pT1, V0,L0,Ro	HCV	−	ND	ND
**24**	57	f	+	II	ypT1, ypN0, L0, V0, R0	HCV	+*	−	−
**25**	58	f	+	II	pT1, pN0, R0	HCV	−	ND	ND
**26**	59	f	+	II	pT1, R0	HCV	−	ND	ND
**27**	62	f	−	II	pT2, pNX, L0, V1, R0	HCV	+	T: − NT: +	−
**28**	67	f	+	I	pT1, R0	HCV	+*	−	−
**29**	67	f	−	II	pT3, pNX, R0	HCV	+	−	−
**30**	69	f	+	I	NA	HCV	+*	−	−
**31**	70	f	NA	NA	NA	HCV	−	ND	ND
**32**	72	f	+	II	pT1, pNX, L0, V0, R0	HCV	+*	T: + NT: +	−
**33**	77	f	−	II	rypT2, pN0, V0, L0, R0	HCV	+	−	−
**34**	81	f	−	II	pT1, pN0, R0,	HCV	+	−	−
**35**	41	m	+	III	NA	HBV	+	−	T: + NT: −
**36**	44	m	+	II	NA	HBV	+	−	−
**37**	53	m	NA	II	pT2, pNX, L0, V1, R0	HBV	−	ND	ND
**38**	55	m	+	II	ypT2, ypNX, L0, V0, R0	HBV	−	ND	ND
**39**	60	m	+	NA	ypT2, ypN0, L0, V0, R0	HBV	−	ND	ND
**40**	62	m	NA	II	pT2, pN0, R0	HBV	−	ND	ND
**41**	76	m	+	I	pT1, pN1	HBV	−	ND	ND
**42**	79	m	+	II	ypT3, ypN1, L0, V0, R0	HBV	+	−	−
**43**	50	f	−	II	pT1, pN0, L0, V0, R0	HBV	−	ND	ND
**44**	50	f	−	II	pT1, pN0, L0, V0, R0	HBV	−	ND	ND
**45**	46	m	+	II	pT1, V0, L0, N0	alcoholic liver disease	+	−	−
**46**	53	m	+	II	pT2, pN0, PM0, R0	alcoholic liver disease	+	−	−
**47**	56	m	+	I	pT1, pNX, L0, V0, R0	alcoholic liver disease	+	T: + NT: −	−
**48**	57	m	+	II	pT1, pN0, R0	alcoholic liver disease	+	−	−
**49**	58	m	NA	II	pT4, R0	alcoholic liver disease	+	−	−
**50**	60	m	NA	II	pT2, pN0, R0	alcoholic liver disease	−	ND	ND
**51**	60	m	+	I	pT1, R0	alcoholic liver disease	−	ND	ND
**52**	64	m	+	II	ypT1, V0, L0, pN0, R0	alcoholic liver disease	−	ND	ND
**53**	65	m	NA	II	pT2, R0	alcoholic liver disease	+	−	T: − NT: +
**54**	65	m	+	II	ypT1, R0	alcoholic liver disease	+*	−	T: + NT: −
**55**	69	m	+	III	pT2, V1, R0	alcoholic liver disease	+*	−	−
**56**	69	m	+	III	pT1, V0, L0, pN0, R0	alcoholic liver disease	+	−	−
**57**	70	m	+	II	ypT1, R0	alcoholic liver disease	+	−	−
**58**	71	m	+	II	pT1, R0	alcoholic liver disease	−	ND	ND
**59**	73	m	NA	II	pT3, N0, R1	alcoholic liver disease	+	T: − NT: +	T: + NT: −
**60**	74	m	+	II	pT1, pNX, L0, V0, R0	alcoholic liver disease	−	ND	ND
**61**	52	f	NA	NA	NA	alcoholic liver disease	+	−	−

*promoter methylation was only detected in non-tumour adjacent tissue.

Abbreviations: f, female; m, male; Meth., methylation; NA, not available; ND, not determined; NT, non-tumour adjacent tissue; T, tumour.

### DNA extraction from formalin-fixed, paraffin-embedded tissue

Formalin-fixed, paraffin-embedded tumour and non-tumour adjacent tissue was taken from all patients investigated. Representative tissue regions were identified by microscopic examination. In all cases, areas of tumour tissue with more than 80% of malignant cells were selected. Areas from 10 slides of 4–5 µm thickness were microdissected using a surgical scalpel. DNA was isolated from the paraffin material using RecoverAll Total Nucleic Acid Isolation Kit (Ambion, Germany) according to the manufacturer's protocol.

### Bisulfite treatment and methylation specific PCR (MSP)

Bisulfite conversion of the purified DNA (1.5 µg) was performed with EpiTectBisulfite Kit (Qiagen) according to the manufacturer's protocol. Success of treatment was negatively controlled by TP53 PCR.

MSP of MLH1, PMS2, MSH2 and p16 was carried out in a volume of 25 µl containing 0.8 µM forward and reverse primer, 0.2 µM dNTPs (each), 2.5 µl buffer B (Invitrogen) and 0.5 µl Tempase Polymerase (Amplicon) using following conditions: 95°C 15 min, 40 cycles of 95°C 30 s, Tm 30 s, 72°C 40 s followed by an end-elongation of 5 min at 72°C. PCR primers and corresponding annealing temperatures (Tms) are listed in Table 2.

**Table 2:** Primer sequences and annealing temperatures used in MSP.

Gene	Primer	Sequences 5′→3′	Tm (C°)	Fragment length (bp)
*MLH1*	MLH1_unmet_fwd	TTTTGATGTAGATGTTTTATTAGGGTTGT	60	124
	MLH1_unmet_rev	ACCACCTCATCATAACTACCCACA		
	MLH1_met_fwd	ACGTAGACGTTTTATTAGGGTCGC	60	115
	MLH1_met_rev	CCTCATCGTAACTACCCGCG		
*PMS2*	PMS2_unmet_fwd	GTAGGTGGGAAGTTTTATATGGAG	60	148
	PMS2_unmet_rev	CCAATCTCCATCATAACCTCTAACA		
	PMS2_met_fwd	AGAGGCGCGTCGTTTTCGTG	60	121
	PMS2_met_rev	CTCCGTCGTAACCTCTAACG		
*MSH2*	MSH2_unmet_fwd	GTTGTTGTGGTTGGATGTTGTTT	60	143
	MSH2_unmet_rev	CAACTACAACATCTCCTTCAACTACACCA		
	MSH2_met_fwd	TCGTGGTCGGACGTCGTTC	60	132
	MSH2_met_rev	CAACGTCTCCTTCGACTACACCG		
*p16*	p16_unmet_fwd	TTATTAGAGGGTGGGGTGGATTGT	60	151
	p16_unmet_rev	CAACCCCAAACCACAACCATAA		
	p16_met_fwd	TTATTAGAGGGTGGGGCGGATCGC	65	150
	p16_met_rev	GACCCCGAACCGCGACCGTAA		
*TP53*	TP53_fwd	TGGGTTGATTCCACACCCC	59	162
	TP53_rev	AACCAGCCCTGTCGTCTCTC		

MSP was positively controlled with DNA of HeLa cells treated with SssI methyltransferase (NEB) prior bisulfite conversion.

PCR products were analysed by 2% agarose gel electrophoresis.

Results of MSP were verified by repeated analysis.

### Analysis of Microsatellite Instability (MSI)

DNA of tumour and corresponding non-tumour adjacent tissues showing promoter methylation in one of the tested MMR genes were investigated for MSI. For that purpose, we analysed two commonly used mononucleotide marker loci, BAT25 and BAT26.

PCR amplification was performed with following primers: BAT25_f (6FAM) TCGCCTCCAAGAATGTAAGT, BAT25_r TCTGCATTTTAACTATGGCTC, BAT26_f (NED)TGACTACTTTTGACTTCAGCC and BAT26_r AACCATTCAACATTTTTAACCC. The PCR reaction contained 1 µM forward and reverse primers, 0.25 µM dNTPs (each), buffer D (Invitrogen) for BAT25 or buffer E (Invitrogen) for BAT26, 0.25 µl AmpliTaqGold Polymerase (Roche). The PCR consisted of an activation of 8 min at 95°C, 45 cycles: 95°C 30 s, 55°C 15 s, 72°C 60 s and ending with an elongation at 72°C for 10 min. The PCR products were controlled by agarose gel electrophoresis and purified with QIAquick PCR Purification Kit (Qiagen, Germany) according to the manufacturer's protocol. 1.5 µl PCR product, 0.5 µl ROX-Standard (Gene Scan 500 ROX Size Standard, Applied Biosystems) and 8.5 µl HIDI formamide (Applied Biosystems) were analysed with GeneMapper and PeakScanner software of the 3130*xl* Genetic Analyzer (Applied Biosystems).

Finally, experiments were repeated several times to ensure reliability of MSI results.

### Statistics

*P* values were determined using Fisher's exact test. All reported *P* values were 2-sided, and *P* values less than 0.05 were considered as statistically significant.

## Results

### Promoter methylation analysis in HCC tissue

**Figure 1:**
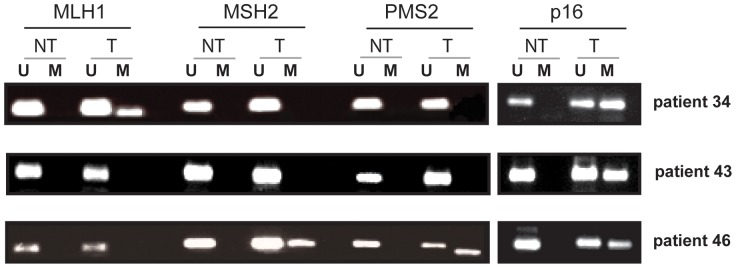
MSP analysis. Representative examples of MSP analysis for MLH1, MSH2, PMS2 and p16 methylation in HCC and non-tumour adjacent tissues were shown. Bisulfite-modified DNA was amplified using MSP primers specific to a CpG-rich region of each gene promoter. PCR-amplified products were resolved by 2% agarose gel electrophoresis. (U) Lanes represent amplification of unmethylated alleles, and (M) lanes contain only methylated alleles.

Promoter methylation of MLH1, PMS2, MSH2 and p16 was analysed by MSP in tumour and non-tumour adjacent tissue of 61 patients with HCC of which 34 were associated with HCV, 10 with HBV and 17 with alcoholic liver disease (Table 1). Representative examples of the MSP are shown in Figure 1.

Our data demonstrate that promoter methylation of MMR genes is associated with HCC. We found that 59% of all patients (36/61) showed CpG island alterations at least in one of the tested MMR genes (Table 1). However, promoter methylation was detectable in tumour but partly also in non-tumour adjacent tissue in 28 cases while 8 patients showed changed CpG pattern only in the non-tumour adjacent tissue (Table 1).

### Distribution of gene promoter methylation

Altogether, 101 promoter methylations were found, whereby the frequency of methylation was most dominant in the tumour and not in the non-tumour adjacent tissue (65 vs. 36) (Table 3).

**Table 3:** Frequency of promoter methylation.

Origin of HCC	MLH1, n			PMS2, n			MSH2, n			p16, n			Frequency of methylation [%]
HCV	U	M	p-value	U	M	p-value	U	M	p-value	U	M	p-value	
**T**	30	4	0.114	31	3	0.709	25	9	1.000	16	18	**0.011**	**19.8**
**NT**	34	0		29	5		26	8		27	7		
**HBV**													
**T**	9	1	1.000	9	1	1.000	9	1	1.000	4	6	**0.057**	**16.3**
**NT**	10	0		8	2		9	1		9	1		
**Alcoholic liver disease**													
**T**	17	0	1.000	12	5	0.688	12	5	1.000	5	12	**0.001**	**25**
**NT**	16	1		14	3		11	6		15	2		
**Σ**		6			19			30			46		

p≤0.05 =  significant.

Abbreviations: NT, non-tumour adjacent tissue; T, tumour.

**Figure 2:**
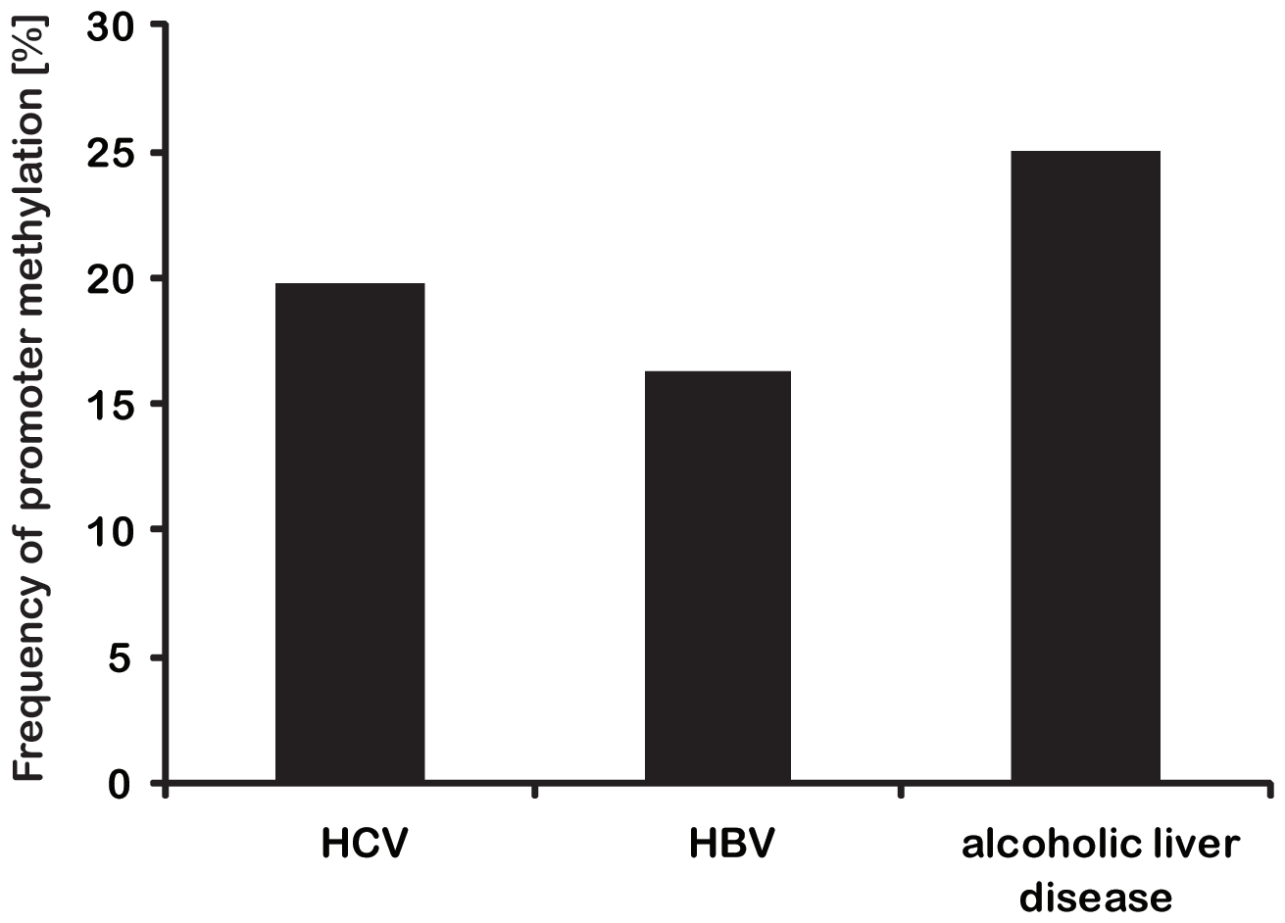
Frequency of promoter methylation. Promoter methylation of MLH1, PMS2, MSH2 and p16 was determined in HCC of different origins. As presented in bar graph, the frequency of methylation in the tested promoters and tissues was 19.8% in HCV infected, 16.3% in HBV infected and 25% in patients with alcoholic liver disease.

Our results did not show a correlation of promoter methylation with different origins of HCCs. The frequency of methylation in all tested promoters and tissues was 19.8% in HCV infected, 16.3% in HBV infected and 25% in patients with alcoholic liver disease (Table 3, Figure 2).

Looking at each gene promoter in detail, 38.5% (46) of methylations were found in p16, 24.6% (30) in MSH2, 15.6% (19) in PMS2 and only 4.9% (6) in MLH1 (Table 3). Thus, p16 and MSH2 were the most frequently affected genes whereupon the alteration of p16 was significant in all HCCs regardless of their origin (p>0.011 (HCV); p>0.05 (HBV); p>0.003 (alcoholic liver disease)) (Table 3).

Interestingly, methylation of MSH2 and PMS2 were similarly distributed in tumour as well as non-tumour adjacent tissues while p16 and MLH1 were most dominantly methylated in the tumour tissue.

### Methylation and clinicopathological association

We also investigated the association between promoter methylation of MLH1, MSH2, PMS2 and p16 and the clinicopathological features of the HCC patients, including age, gender, pathological grade as well as pathological stage (Table 4).

**Table 4:** Relationship between promoter methylation and clinicopathologic parameters of HCC patients (N = 61).

Characteristics	MLH1, N				PMS2, n				MSH2, n				p16, n			
	U	M	p-value	Frequency of methylation [%]	U	M	p-value	Frequency of methylation [%]	U	M	p-value	Frequency of methylation [%]	U	M	p-value	Frequency of methylation [%]
**Age (Years)**																
**≤60**	27	5	0.198	15.6	25	7	0.767	21.9	25	7	0.100	21.9	14	18	0.601	56.3
**>60**	28	1		3.4	21	8		27.6	16	13		44.8	10	19		65.5
**Sex**																
**Male Female**	41 14	4 2	0.647	8.9 6.7	34 12	11 4	1.000	24.4 25	30 11	15 5	1.000	33.3 31.3	17 7	28 9	0.768	62.2 56.3
**Grade** *																
**NA**	4	2		33.3	5	1		16.7	6	0		/	4	2		33.3
**I**	7			/	4	3		42.8	5	2		28.6	2	5		71.4
**II**	38	4		9.5	31	11		26.2	29	13		30.9	18	24		57.1
**III**	6	0		/	6	0		/	1	5		83.3	0	6		100
**Stage** *																
**NA**	8	2		20	8	2		20	6	4		40	4	6		60
**pT1**	24	2		7.7	18	8		30.8	19	7		26.9	11	15		57.7
**pT2**	13	1		7.1	12	2		8.3	11	3		12.5	7	7		50
**pT3**	7	1		12.5	5	3		37.5	3	5		62.5	1	7		87.5
**pT4**	3	0		/	3	0		/	2	1		33.3	1	2		66.7

p≤0.05 =  significant.

Abbreviations: M, methylated; N, number of patients; NA, not available; U, unmethylated.

*To stratify patients, the latest AJCC TNM classification was used [28].

The proportion of patients who generated HCC≤60 years vs. those who were diagnosed for HCC>60 years was similar (32 (52.5%) cases vs. 29 (47.5%) cases) and the frequency of promoter methylation in both groups was without relevant difference in any of the analysed genes (Table 4).

Although the amount of analysed HCC tissue of female patients was only 1/3 compared with the HCC tissue of male patients (16 females (26.2%) vs. 45 males (73.8%)) there was no significant difference in the frequency of promoter methylation in both groups (Table 4).

Looking at the relationship of promoter methylation status and the corresponding tumour stage our data show an increase of methylation in correlation to advanced tumour stage in all considered genes (Table 4).

However, the frequency of promoter methylation of p16 and MSH2 was higher than that of MLH1 or PMS2 in general and highest in HCCs of grade II+III and stage pT3 (Table 4).

### MSI analysis

Paraffin-embedded HCC and the corresponding non-tumour adjacent tissue were investigated for MSI of all those samples showing promoter methylation in one of the tested MMR genes using two mononucleotide markers, BAT25 (a poly(A) tract occurring in intron 16 of c-kit [18]) and BAT26 (a poly(A) tract localized in the fifth intron of hMSH2 [19]). Both markers are extremely sensitive and specific and are commonly used for MSI analysis of Lynch syndrome [20].

**Figure 3:**
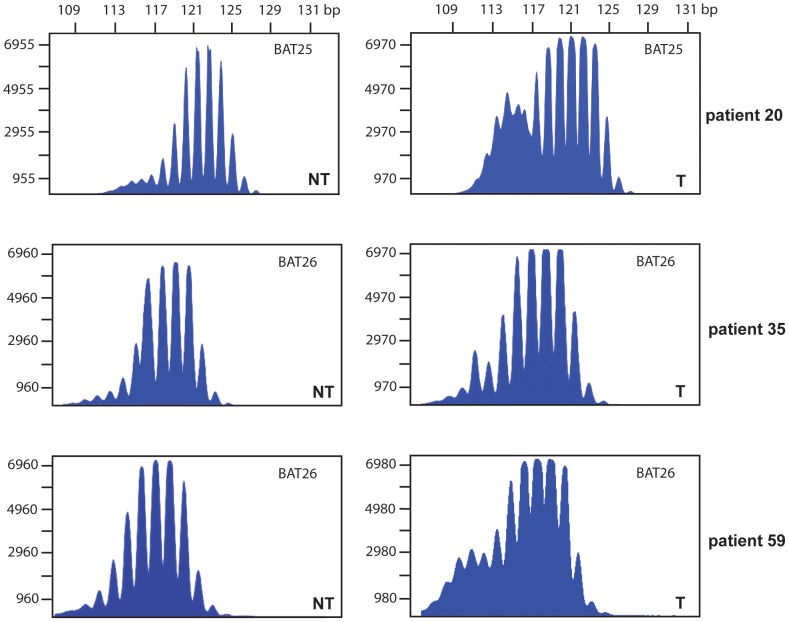
MSI analysis. Analysis of commonly used mononucleotide MSI marker loci BAT25 and BAT26 was performed in all patients showing promoter methylation in one of the tested MMR genes. Three examples are shown here. (T) HCC tissue, (NT) non-tumour adjacent tissue.

MSI of BAT25 could be determined in 8 patients: 4 generated MSI in tumour tissue, 2 in tumour as well as non-tumour adjacent tissues and 2 showed MSI only in non-tumour adjacent tissue (Table 1). MSI of BAT26 was detectable in 5 cases: 4 generated MSI in tumour tissue and one showed MSI in non-tumour adjacent tissue (Table 1). MSI of BAT25 is exemplarily shown in Figure 3 for three cases.

## Discussion

In this study, we determined the promoter methylation status of four different tumour suppressor genes in a cohort of 61 European HCC patients to further investigate controversial data about possible underlying mechanisms for changed promoter methylation in HCCs [7], [8], [9], [10], [11], [12], [13], [14]. Our data demonstrate that HCCs associated with different viral aetiologies or alcoholic liver disease show frequently aberrant promoter methylation pattern of the MMR genes MLH1, MSH2 and PMS2 or the cell cycle regulator gene p16, but a significant correlation with viral infection could not be observed.

Our observations are in accordance to Zhang et al. who analysed promoter methylation of MLH1, MSH2 but also MSH3 in 38 HCC cases of different origins and showed that hypermethylation of MMR genes is a common phenomenon in HCCs [21]. Moreover, Matsukura and co-workers tested a cohort of 46 HCCs and found silencing of MLH1 in HCV, HBV as well as non-virus associated HCCs [8]. However, we detected promoter methylation not only in tumour but also in non-tumour adjacent tissue. In line with this, Helal et al. detected reduced MLH1 and MSH2 expression in HCCs but also in adjacent non-cancerous surrounding tissues [22]. Possibly, the used tumour surrounding tissue already contained precancerous cells in some cases which might be detectable by high sensitive MSP analysis.

Looking at our methylation data in detail, the highest frequency of methylation was detected in MSH2 as well as in p16 preferentially in pathological T3 stages. This is consistent with Wani and co-workers who detected reduction of MMR protein expression especially in advanced-stage HCCs [23] and Zhang et al. who determined MSH2 to be most often affected in the analysed cohort [21].

However, some groups emphasised a close correlation between HCC associated with HCV infection and reduced expression or aberrant promoter methylation of MMR genes [8], [24], especially p16 alterations were postulated to be HCV associated [10], [16]. Others described the influence of HCV on DNA methyltransferase 1 [25], [26] a protein essential for the regulation of MLH1 and MSH2 expression [27]. Since chronic HCV infection is a major risk factor for HCC and HCC samples of HCV infected patients were only [22] or most dominantly analysed [8], [24] in the cited publications we rather hypothesize that promoter methylation of MMR proteins and p16 is frequently occurring during HCC progression in general without close connection to HCV.

To strengthen our data on detected promoter methylation of MMR genes which results in protein loss and function we additionally analysed MSI. However, MSI could only partly be found at BAT25 as well as BAT26. One possible explanation for the low detection of MSI might be the problem of general weak proliferation rates of hepatocytes, a basic requirement for MSI development, which was observed and discussed by Wani et al. [23]. In line, Wang et al. could not detect any MSI at BAT26 after determining strong reduction of MMR protein expression in their HCC collective [14].

## Conclusions

In summary, we could show that promoter methylation of essential tumour suppressor genes is detectable in HCCs most dominantly in advanced tumour stages but there was no correlation to an underlying HCV infection. However, since loss of MMR proteins was previously described to be not only responsible for tumour development but also for chemotherapy resistance, the knowledge of mechanisms jointly responsible for HCC progression might enable significant improvement of individual HCC therapy in the future.
